# Comparison Between Expression Microarrays and RNA-Sequencing Using UKBEC Dataset Identified a *trans*-eQTL Associated with *MPZ* Gene in Substantia Nigra

**Published:** 2020-09-16

**Authors:** Letitia M.F. Sng, Peter C. Thomson, Daniah Trabzuni

**Affiliations:** 1 The University of Sydney, School of Life and Environmental Sciences, Australia; 2 Department of Neurodegenerative Disease, UCL Queen Square Institute of Neurology, United Kingdom; 3 Department of Genetics, King Faisal Specialist Hospital and Research Centre, Saudi Arabia

**Keywords:** neuroscience, expression QTL, MPZ gene, RNA-Seq, human brain

## Abstract

In recent years, the advantages of RNA-sequencing (RNA-Seq) have made it the platform of choice for measuring gene expression over traditional microarrays. However, RNA-Seq comes with bioinformatical challenges and higher computational costs. Therefore, this study set out to assess whether the increased depth of transcriptomic information facilitated by RNA-Seq is worth the increased computation over microarrays, specifically at three levels: absolute expression levels, differentially expressed genes identification, and expression QTL (eQTL) mapping in regions of the human brain. Using the United Kingdom Brain Expression Consortium (UKBEC) dataset, there is high agreement of gene expression levels measured by microarrays and RNA-seq when quantifying absolute expression levels and when identifying differentially expressed genes. These findings suggest that depending on the aims of a study, the relative ease of working with microarray data may outweigh the computational time and costs of RNA-Seq pipelines. On the other, there was low agreement when mapping eQTLs. However, a number of eQTLs associated with genes that play important roles in the brain were found in both platforms. For example, a *trans*-eQTL was mapped that is associated with the *MPZ* gene in the substantia nigra. These eQTLs that we have highlighted are extremely promising candidates that merit further investigation.

## Introduction

Gene expression analyses such as differential expression (DE) analysis were developed to understand the patterns of gene expression which in turn can serve as indicators of biological activity. Recently, gene expression data have been integrated with genomic data in the form of expression quantitative trait loci (eQTL) analyses, providing further insights into disease aetiology and complex regulatory networks [1,2].

High-throughput microarray technology has facilitated large-scale gene expression studies, especially as the cost of arrays decrease while becoming more flexible in their design. Another added benefit of microarray technology is that the analysis of microarray expression data is relatively straightforward, as the output from such analyses is in the form of continuous numerical measurements of probe intensities [3]. However, its reliance on hybridisation limits measurements to known transcripts and background noise reduces the accuracy of gene expression measurements [4]. Currently, RNA-Sequencing (RNA-Seq) technology is a sequencing-based method that has risen to become the most popular technology for measuring gene expression. This is in part due to its large dynamic range and high reproducibility [5]. Furthermore, RNA-Seq allows for de novo assembly and detection of expression at multiple levels including isoforms and splice junctions, providing more details of the transcriptome [6].

RNA-Seq presents many bioinformatical challenges and requires many computationally costly steps and results in integer counts for each genomic feature of interest [7,8]. Therefore, many RNA-Seq data pipelines were developed to model the count data using discrete distributions such as the negative binomial and the Poisson which have less mathematical tractability than the normal distribution [3].

To date, there have been a limited number of studies [9,10] on how the two technologies compare in the human brain which is important given that gene expression patterns have been shown to be tissue-specific [11]. In addition, to our knowledge, there has been no study looking at how the two technologies compare at three levels: absolute expression levels, differentially expressed gene (DEG) detection and eQTL identification for the same dataset.

This study therefore set out to assess the agreement between microarrays and RNA-Seq technology at these three levels in the human brain using genotype, microarray, and RNA-Seq expression data from two brain regions, the putamen (PUTM) and substantia nigra (SNIG) available as part of the United Kingdom Brain Expression Consortium (UKBEC) dataset.

## Materials and Methods

All bioinformatics and statistical computing were completed in the R environment v 3.6.0 [12]. Due to the computationally-intensive nature of working with RNA-Sequencing including alignment and quantification, some work was performed using The University of Sydney’s High-Performance Computing cluster, Artemis.

### Quantification of expression data

#### Microarrays (UKBEC)

The generation of the gene expression array data are as described by Trabzuni, et al. [13] followed by Ramasamy, et al. [14]. Briefly, RNA was extracted from ten regions of 134 neurologically normal human brain samples of Caucasian descent and processed using Affymetrix Human Exon 1.0 ST arrays. Robust multi-array average (RMA) normalisation [15] was applied on all arrays across all regions followed by a log2 transformation. Gene-level expression values were calculated using the Winsorized mean expression of all probe sets corresponding to 26,493 genes, as identified by using Netaffx annotation file Release 31 (HuEx-1_0-st-v2 Probeset Annotations). The resulting data were residual-adjusted for brain bank, gender and batch effects.

#### RNA-sequencing

The same two regions (PUTM and SNIG) from the same RNA samples as the microarray study were selected for the RNA-seq platforms. However, due to difficulties in sample collection, instead of 134 samples, there was a total of 114 samples of which 62 samples had both PUTM and SNIG regional data. The remaining samples had data from only one region: 7 from SNIG and 45 from PUTM which ultimately, resulted in a total sample size of 107 for PUTM and 69 for SNIG.

Briefly, of these brain samples, RNA-seq library preparation and sequencing were prepared by the UKBEC in conjunction with AROS Applied Biotechnology. In summary, 100 ng total RNA was used as input for cDNA generation using the NuGen’s Ovation RNA-Seq System V2 (NuGen Technologies). The RNA was processed according to the manufacturer’s protocol resulting in amplified cDNA from total RNA and concomitant de-selection of rRNA. Notably, reverse transcription in this protocol was carried out using both oligo(dT) and random primers. This allowed total RNA profile patterns to be assessed with the latter and locations of splicing to be inferred. Next, 1 μg of the cDNA was fragmented using a Covaris S220 Ultrasonicator and the fragmented cDNA was used as the starting point for Illumina’s TruSeq DNA library preparation. Finally, library molecules containing adaptor molecules on both ends were amplified through 10 cycles of PCR. The libraries were sequenced using Illumina’s TruSeq V3 chemistry/HiSeq2000 and 100 base pair paired-end reads. The sequencing data were then converted to FASTQ files using Illumina’s CASAVA Software. For more details, refer to Guelfi, et al. [16].

The generated FASTQ files of paired-end reads were put through the FastQC program (version 0.11.8) for quality control [17]. Some samples were sequenced again due to poor quality and based on FASTQC reports, either the original FASTQ or re-sequenced FASTQ files were used for further analysis.

The Rsubread [18] workflow was used to quantify read counts. Firstly, an index was built from the human genome (hg19/GRCh37, release 31) using *Rsubread :: buildindex* and reads from each sample were than mapped to this reference using *Rsubread :: align* resulting in BAM files. The resulting alignments were then assigned at the gene-level based on the same annotations used for the microarray as described above. This was to reduce variability between technologies and to allow comparability. This resulted in raw read counts for 26,493 genes across the genome.

These counts were then put through the edgeR-limma workflow as described by Law, et al. [19] for the necessary adjustments including transformations and normalisations as well as exploratory plotting. Notably, raw counts were transformed to log2-counts per million (log-CPM) to adjust for differing library sizes across samples. Lowly expressed genes were then filtered out using *edgeR :: filterByExpr* where genes with a log-CPM ≥ 1.0945 across the minimum group sample size (i.e. SNIG=69 samples) were kept. This reduced the number of genes to 17,485 (66% of original number of genes). Trimmed mean of M-values (TMM) normalisation was then applied but the effect was slight given that scaling factors were close to 1.

*Voom* was then applied on the resulting read counts to obtain log-CPM gene expression values with associated weights [3]. This method then allowed linear modelling to be used by incorporating the calculated precision weights which has been shown to perform just as well as count-based methods developed for RNA-Sequencing [7].

#### Assessing agreement between platforms

The 17,485 genes that were kept after the filtering step in the RNA-Seq data pre-processing stage were subsetted out of the microarray data. Furthermore, as only the RNA from some samples were sequenced from PUTM and SNIG (62 samples), the corresponding samples from the two regions were subsetted out from the microarray data as well. This was to allow for comparability across the technologies.

#### Correlation of absolute expression levels

Spearman correlations between normalized microarray expression data and the *voom*-transformed RNA-Seq expression data were calculated across the samples for each region separately, i.e. each correlation was based on 17,485 gene expression pairs. A linear mixed model was then fitted to investigate the effect of age, gender, cause of death, region and post-mortem interval (PMI) on the calculated correlation coefficients. Random brain sample effects were included to allow for the paired data (PUTM and SNIG) when they occurred. Note that the correlations were transformed using Fisher’s *Z*-transformation and splines were applied to the continuous explanatory variables (Age and PMI) to allow for a possible nonlinear effect on the correlation. This analysis was undertaken using the commercial R package ASReml-R [20].

### Differentially Expressed (DE) genes

The analysis method described in Trabzuni, et al. [21] was applied to the microarray expression data and the *voom* RNA-Seq expression data to classify DEGs. The 17,485 genes across the two regions (PUTM and SNIG) were used to fit a linear mixed model (LMM) to assess sources of variation at an overall level. Again, this was analysed using the commercial R package ASReml-R [20]. The form of the model was:

$$\begin{array}{c} y_{ijk}=\mu +Gene_{i}+Gene.Region_{ij}+\varepsilon_{ijk} \end{array}$$

where *y_ijk_* = microarray/*voom* transformed RNA-Seq expression data as described above for brain sample *k; μ* = overall mean expression value; *Gene_i_* = random effect due to gene, assumed $N\left(0,\sigma_{G}^{2}\right)$; *Gene. Region_ij_* = random effect due to gene *i* in a region *j*, assumed $N\left(0,\sigma_{GR,j}^{2}\right)$; *j* = 1,2; and *ε_ijk_* = random error, assumed $N\left(0,\sigma_{\varepsilon }^{2}\right)$. Note that this fits a heterogenous variance model for the gene × region random effects to allow for different variances between the two regions and that *voom*-calculated precision weights at the observation level were added to the linear mixed model for RNA-Seq data. Because of the different measurement scale of microarray vs RNA-Seq data, levels of variation between platforms were compared using coefficients of variation, (σ/μ) ×100.

The difference between the two regions (PUTM – SNIG) using the best linear unbiased predictions (BLUPs) of the gene × region random effects were then obtained and modelled as a finite mixture distribution of DE vs non-DE genes and fitted using the expectation-maximisation (EM) algorithm [22]. The posterior probability (τ_i_) of each gene being DE was returned and a threshold of τ_i_ ≥ 0.8 was used to determine DE status of each gene. This is similar to the method described by Trabzuni, United Kingdom Brain Expression and Thomson [21].

#### Single nucleotide polymorphism genotype data

Overall, nearly 1 million SNPs were genotyped on the Illumina Infinium Omni1-Quad BeadChip array. The first filter of the major allele frequency (MAF)>5% reduced the SNP set to 787,220. Following this, any SNP that was missing in any of the sample was omitted leaving a final set of 720,851 SNPs. Linkage disequilibrium (LD) analysis was then carried out and haplotype blocks were identified using a threshold of R^2^>0.5.

### Expression Quantitative Trait Loci (eQTLs)

The R package MatrixEQTL [23] was used as a computationally efficient method to detect eQTLs with the microarray data using simple linear regressions, as was used in previous analyses of the UKBEC dataset [14,24]. Microarray expression values were regressed on SNP genotypes (encoded as dosages of the minor SNP allele) as an additive model without covariates. The resultant estimated regression coefficients were the effect sizes of each SNP-gene pair. As part of the MatrixEQTL package, an adjusted Benjamini-Hochberg false discovery rate (FDR) [25] was used to control for multiple testing and a threshold of FDR<0.01 was used to determine significant eQTLs. This procedure was run separately for each brain region.

Similarly, *voom*-transformed RNA-Seq expression data were regressed on SNP genotypes separately for each region. However, due to the observation-level weights that need to be included in the linear model, MatrixEQTL could not be used. Instead, R code was written using the *lm* function from the base R package that allows for specific weights. The same adjusted Benjamini-Hochberg FDR was then calculated and the threshold of 0.01 was used to determine significant RNA-Seq eQTLs. Note the need for an adjusted FDR was because a preliminary *p*-value threshold of 1 × 10^-4^ was applied before FDR was calculated. This was to decrease the size of the output files.

## Results

Results show that the average Spearman correlations between microarray expression data and the *voom*-transformed RNA-Seq expression data calculated for each region across samples were moderately high (PUTM: 0.669, SNIG: 0.658) (see Supplementary Materials for correlations across transcripts). This result is consistent with previous studies of the human brain and of different tissues [9]. Furthermore, there was no significant effect of region (*p*=0.17), age (*p*=0.59), PMI (*p*=0.65), gender (*p*=0.30) or cause of death (*p*=0.58) on the correlation between platforms. This finding was expected given that the RNA material was extracted from the same individuals and both RNA-Seq and microarray data underwent extensive quality control measures. However, it is still important to note that these variables did not play a significant role for this dataset, and therefore can be disregarded as possible factors for the high correlations calculated across the samples.

A comparable number of DEGs were found using microarray data (1553) and RNA-Seq data (1632) with 1002 DEGs identified in both platforms. Encouragingly, both platforms agreed on the direction of the effects of these 1002 DEGs. This is unsurprising given the high Spearman’s correlation between the absolute microarray expression and RNA-Seq expression levels. These findings suggest that the relative ease of using microarrays may outweigh the computational time and cost of RNA-Seq pipelines for quantifying absolute expression levels or identifying DEGs in the regions of the human brain. Although, this would also depend on other qualifications of the study including using only known transcripts.

The total number of significant eQTLs (FDR ≤ 0.01) found across both regions using microarray expression data was much lower compared to using RNA-Seq data (Microarray: 579, RNA-Seq: 1821). Interestingly, before the FDR threshold was applied (i.e. *p*-value ≤ 10^-4^) more eQTLs were identified using microarray data (PUTM: 2,121,169; SNIG: 2,220,017) than RNA-Seq data (PUTM: 1,249,909; SNIG: 1,434,729). Furthermore, in this study we considered that eQTLs identified as significant by both platforms are true eQTLs and we classified them into two categories as described in Sng, Thomson and Trabzuni [24] to provide more biologically interesting insights: (i) multi-region if eQTL in both PUTM and SNIG (MR-eQTLs) or (ii) single-region if found in either region only (SR-eQTL). They were then further separated into *cis*- and *trans*-eQTL distinctions.

**Figure 1:**
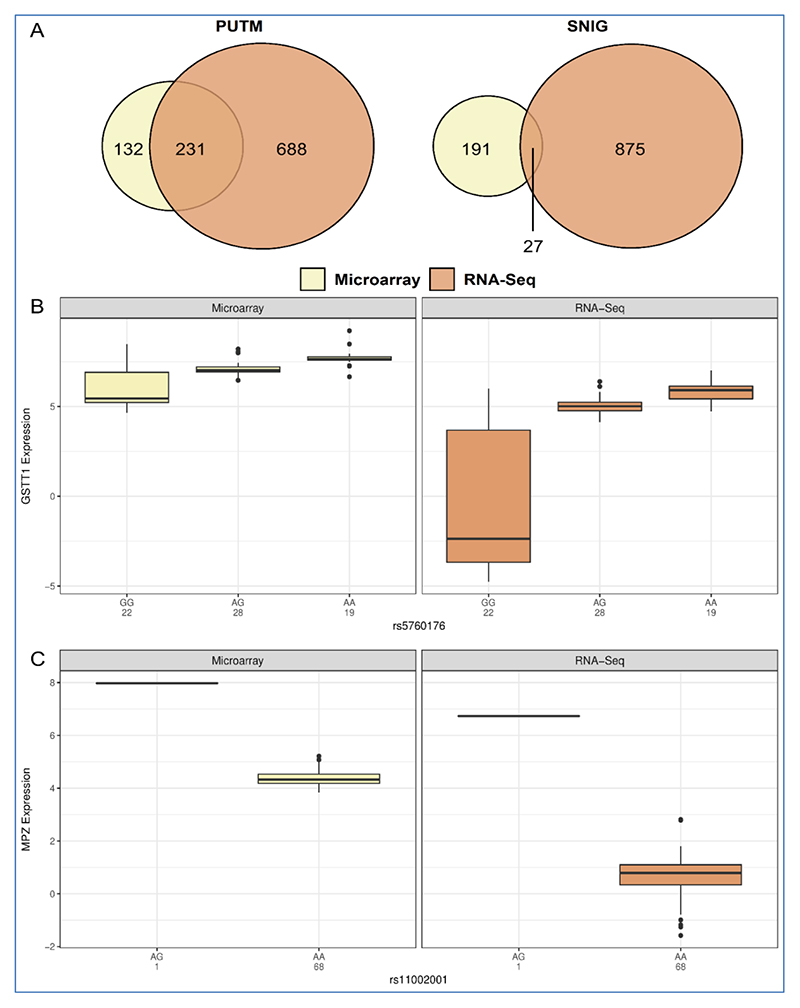
(A) Significant eQTLs (FDR ≤ 0.01) identified by microarrays against RNA-Seq in PUTM and SNIG. There is a much higher number of eQTLs in common in PUTM than SNIG. Overall, RNA-Seq data identified more significant eQTLs than microarray for both regions. (B) Boxplots of the effect of SNP rs5760176 on *GSTT1* expression levels in PUTM. The AA homozygous genotype is associated with an increase in *GSTT1* expression for both microarray and RNA-Seq data. Note that this eQTL was significant in PUTM only (C) Boxplots of the effect of SNP rs11002001 on *MPZ* expression levels in SNIG. The AA homozygous genotype is associated with a decrease in *MPZ* expression for both microarray and RNA-Seq data. Note that this eQTL was significant in SNIG only and because of sample subsetting for platform comparison, there were no samples with the GG genotype. It is worth mentioning that the GTEx dataset had only one sample with the GG genotype out of 483 samples and in the 1000 Genome dataset, the G allele has a frequency of 0.0136).

There were 231 eQTLs identified by both platforms for PUTM and 27 for SNIG (Figure 1A), i.e. true eQTLs. The small number of eQTLs in common detected in SNIG may be a side effect of the smaller number of eQTLs found by using microarray data from SNIG. These proportions were similar even when mapping to haplotypes instead of individual SNPs and when using a less stringent FDR threshold of 0.05 (see Supplementary Material). It is worth mentioning that due to the need to subset samples for platform comparison, the smaller number of samples from the SNIG may have led to the smaller number of eQTLs detected. There were two *trans*-eQTLs found in both platforms and both were SR-eQTLs identified in SNIG. Additionally, 13 true eQTLs were identified in both regions and all were classified as *cis.* Although the numbers are small, this pattern recapitulates the findings of Sng, Thomson and Trabzuni [24] and GTEx Consortium [1] where *trans*-eQTLs tended to be SR-eQTLs. The majority of the true eQTLs (89.0%) were identified as SR-eQTL in PUTM.

## Discussion

Optimistically, of the true *cis*-eQTLs in the PUTM, some were previously reported to be significant using only microarray data with inferences to brain functions [24]. For example, the increased expression of the gene glutathione S-transferase theta 1 (*GSTT1*) (Microarray: *p*=8.95 × 10^-12^, RNA-Seq: *p*=1.16 × 10^-11^) due to the eQTL rs5760176 (minor allele A) (Figure 1B) was suggested to play a protective role against oxidative stress in the cell which may result in a decreased risk of brain tumours [26]. Another example found is eQTL rs823118 (minor allele T), which was shown to increase gene expression of non-coding RNA *LOC284581* (Microarray: *p*=9.96 × 10^-11^, RNA-Seq: *p*=8.44 × 10^-11^) (see Figure S7). This eQTL has been validated by the GTEx dataset in the same region. The SNP rs823118 has been associated with Parkinson’s disease (PD) and has been associated with expression and methylation patterns in the *NUCKS1-RAB29-PM20D1* region [27,28]. The gene *RAB29* in this locus has been functionally linked to *LRRK2* as a substrate [29,30]. Furthermore, *LOC284581* is located nearby *PM20D1* which suggests that future PD studies should extended the region to *NUCKS1-RAB29-PM20D1-LOC284581.* Taken together, the substantial implications of these eQTLs make them extremely promising candidate genes and pathways for future biological investigations.

The eQTL that we would like to highlight is the *trans*-eQTL identified by both platforms. This *trans*-eQTL (SNP rs11002001) was found in SNIG and was paired with myelin protein zero (*MPZ*) (Microarray: *p*=3.08 × 10^-19^, RNA-Seq: *p*=5.19 × 10^-11^) and retinol dehydrogenase 10 (*RDH10*) (Microarray: *p*=1.28 × 10^-14^, RNA-Seq: *p*=3.21 × 10-10). Specifically, the A minor allele of this eQTL was associated with a decrease in both *MPZ* (Figure 1C) and *RDH10* expression (see Figure S8).

We found that both these gene are reported as eGenes (genes with expression affected by eQTLs) in the brain in the GTEx dataset. The *MPZ* gene is specifically expressed in Schwann cells of the peripheral nervous system and encodes a Type I transmembrane glycoprotein that is a major structural protein of the peripheral myelin sheath. The encoded protein contains a large hydrophobic extracellular domain and a smaller basic intracellular domain, which are essential for the formation and stabilization of the multilamellar structure of the compact myelin. Although this SNP has not been associated with any disease pathology and is in an intergenic region, this result suggests that this eQTL should be investigated further considering the fact that the Schwann cell is a neurilemma cell that produces the myelin sheath around neuronal axons and *MPZ* is associated with Charcot-Marie-Tooth disease, demyelinating, Type 1b [31,32]. Furthermore, a recent study suggests that rs11002001 is a genetic variable associated with an individual response to a specific antidepressant medication [33].

An important limitation in this study was the smaller than ideal minimum sample size advised for eQTL mapping due to the subsetting of samples from the UKBEC dataset. However, the sample size of the current study is still larger than the sample sizes (n ≤ 50) which were shown to lead to unreliable results [24]. There is also the implication of low power to detect eQTLs due to the small sample size but as shown by our previous study [24], sensitivity to detect eQTLs is overall very low. Also, the generalisability of our results to other tissue types and even other brain expression databases is limited as this study focussed on only two regions of the human brain. Further work including more samples and tissue types is required to examine if patterns found in this study extend to other tissue types. A key issue that was raised in this study was the low agreement between platforms when detecting eQTLs. This is despite the high agreement when detecting DEGs and the high correlation between expression values. A possible explanation for this is that although the expression values from each expression platform were highly correlated, they were not identical, and this difference was sufficient to map distinct eQTLs between platforms. Furthermore, from a biological point of view, on the one hand these results provide us with potential and interesting candidate eQTLs (SNPs/transcripts), so we can prioritize them; on the other hand wet laboratories experimental studies is essential to validate, dissect and investigate these results at biological functional and mechanism levels.

Despite the exploratory nature, this study has provided a comprehensive look at how mRNA expression levels measured by microarray and RNA-Seq technologies compare in two regions of the human brain and it demonstrates a high agreement between the two platforms at the absolute expression level and classifying DEGs. More importantly, this study identified a promising *trans*-eQTL associated with the neurologically important gene MPZ as a strong candidate in addition to the previous reported *cis*-eQTLs associated with *GSTT1* and *NUCKS1-RAB29-PM20D1* locus. The authors are aware that trans-eQTLs tend to be taken as false positives by the majority of researchers due to the low replication of *trans*-eQTLs across studies [1] and that our definition of *trans*-eQTLs is based on patterns of observed data [24] rather than any biological classification. However, in the present study, there is high confidence in the validity of this *trans*-eQTL given that it was mapped using both platforms: microarray and RNA-Seq, and significant in GTEx dataset as well. Further validation with other human brain datasets is needed and a case versus control study would be essential.

## Supplementary Material

Supplementary Material
