# Supplementary material for: Comparison Between Expression Microarrays and RNA-Sequencing Using UKBEC Dataset Identified a trans-eQTL Associated with MPZ Gene in Substantia Nigra
Source: Front Neurol Neurosci Res. Author manuscript; Available in PMC 2021 Jul 27. (PMC7611373)
Supplement: Supplementary Material [file EMS116075-supplement-Supplementary_Material.pdf]

Frontiers in Neurology and Neuroscience Research
Volume 1
Article ID: 100001

Research Article

Comparison Between Expression Microarrays and RNA-Sequencing Using UKBEC Dataset Identified a *trans*-eQTL Associated with *MPZ* Gene in Substantia Nigra

Letitia M.F. Sng[1], Peter C. Thomson[1] and Daniah Trabzuni[2,3]*

[1]The University of Sydney, School of Life and Environmental Sciences, Australia

[2]Department of Neurodegenerative Disease, UCL Queen Square Institute of Neurology, United Kingdom

[3]Department of Genetics, King Faisal Specialist Hospital and Research Centre, Saudi Arabia

***Corresponding author**: Daniah Trabzuni, Department of Neurodegenerative Disease, Wing 1.2 (first floor) Cruciform Building, Gower Street, London, WC1E 6BT, UK, Tel: +447872608992; E-mail: d.trabzuni@ucl.ac.uk

Received: August 10, 2020; **Accepted**: September 09, 2020; **Published**: September 16, 2020

Copyright: ©2020 Sng MLF. This is an open access article distributed under the Creative Commons Attribution License, which permits unrestricted use, distribution, and reproduction in any medium, provided the original work is properly cited.

Additional Results

Absolute expression levels

As covered briefly in the main paper, there were moderate Spearman's correlations between the absolute expression levels output from the expression microarrays and the *voom*-transformed RNA-Seq expression for each region. **Error! Reference source not found.** shows the correlation across transcripts instead of samples. There is a wider spread of correlations across transcripts, but the majority still show moderate to high correlation. Interestingly, the transcripts with the lowest correlations are not the same between regions. For example, the transcript with the lowest correlation in PUTM is *MRAP* with -0.314 but in SNIG, it is *FBXW8* with -0.367.

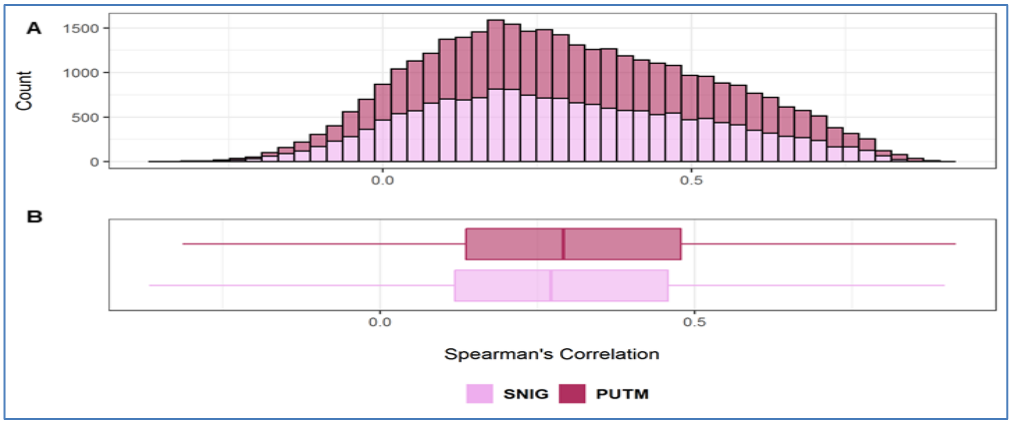

Figure S1. Distribution of Spearman correlations between microarray and RNA-Seq expression levels for each sample for PUTM and SNIG. Both the (A) histogram and (B) boxplot skew towards the higher values but with only a few low outliers especially obvious in SNIG.

Differentially Expressed Genes (DEGs)

We have included more results from the assessment of the agreement of DEGs here. Specifically, more details on the linear mixed model and finite mixture model that was used to identify DEGs as outlined in the materials and methods section above.

The estimates of the overall expression mean (μ) and the variance components from each random effect of the linear mixed model for both microarray and RNA-Seq expression data are shown in Supplementary Table S1.

Table S1. Parameter estimates with their standard errors for linear mixed model fit to the microarray and RNA-Sequencing expression data including their corresponding coefficient of variation (σ/μ) × 100. Clearly, there is higher variation using data from RNA-Seq compared to the microarray in all parameters estimates

Parameter	Microarray		RNA-Seq	
μ	5.876 ± 0.001		3.876 ± 0.018	
$\hat{\sigma}_G^2$ **Gene**	1.657 ± 0.018	21.9%	5.225 ± 0.058	59.0%
$\hat{\sigma}_{GR,1}^2$ **PUTM**	0.119 ± 0.004	5.9%	0.230 ± 0.015	12.4%
$\hat{\sigma}_{GR,2}^2$ **SNIG**	0.049 ± 0.004	3.8%	0.482 ± 0.016	17.9%
$\hat{\sigma}_\varepsilon^2$ **Residual**	0.135 ± 0.0001	6.3%	1.233 ± 0.001	28.6%

It is apparent that RNA-Seq data had larger estimated variances compared to the microarray data. Specifically, the coefficients of variation for all parameter estimates were larger in the RNA-Seq data compared to the microarray data: $\hat{\sigma}_G^2$ (between-gene): +37.1%; $\hat{\sigma}_{GR,1}^2$ (PUTM gene-specific effects)=+6.5%; $\hat{\sigma}_{GR,2}^2$ (SNIG gene-specific effects)=+14.1%. Strangely, the variance (and coefficient of variation) for gene-specific effects in SNIG was higher than that of PUTM in the RNA-Seq data but the opposite was observed for the microarray data.

Taken together, these observations suggest that RNA-Seq technology is more sensitive than microarray technology as the RNA-Seq variance component estimates were relatively larger compared to those from the microarray data, given that the true variability of the effects are equal between the two technologies. On the other hand, another source of this difference could be due to the normalisation techniques or any of the statistical/bioinformatical steps taken on the RNA-Seq counts. Despite these differences in variation, the smoothed scatter plot (Figure S) and calculated Spearman's correlation (r) of the gene-specific effects between the two technologies for each region show high agreement (PUTM: 0.841, SNIG: 0.837).

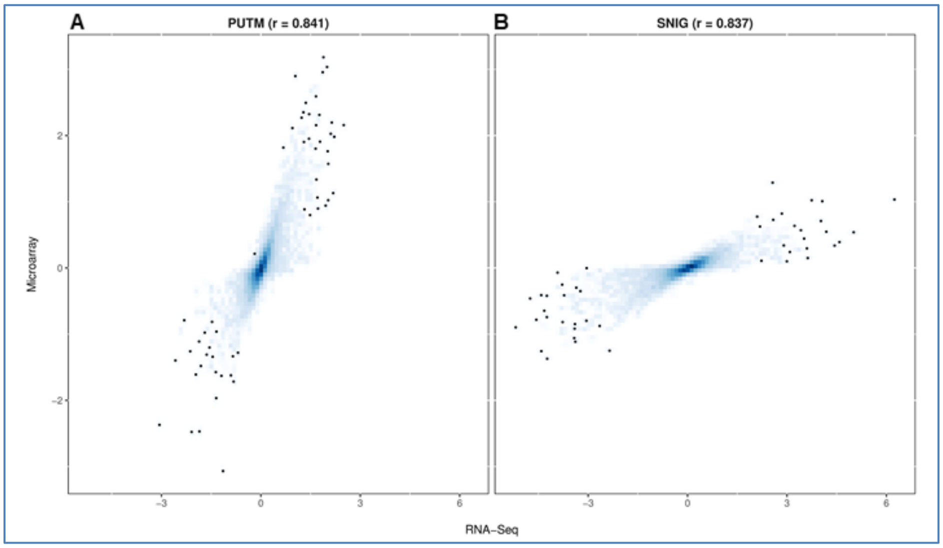

Figure S2. Smoothed scatter plots of the estimated Gene × Region effects for RNA-Seq (*x*-axis) and Microarray (*y*-axis) for (A) PUTM and (B) SNIG. There is a high level of correlation of estimated Gene x Region effects between the two technologies in both brain regions.

We looked at the differences between region-specific gene effects (i.e., differential expression between PUTM and SNIG) for each technology next. Figure S3 shows evidence of upregulated and downregulated differential gene expression between the two regions for both platforms.

Mixture Q-Q plots indicated that the two-component mixture model was adequate for microarray and RNA-Seq expression data and the model parameter estimates for each technology are summarised in Table S2. The calculated

probability that a randomly selected gene shows differential expression was comparable across the two technologies with RNA-Seq having a 0.012 higher probability. As expected, there were higher standard deviations for DEGs than non-DEGs for both technologies (4.33× greater on average). It is worth noting that the standard deviations of both DEG and non-DEGs were higher in RNA-Seq data compared to microarray data which is likely due to the differences in REML-estimated variances that were highlighted previously.

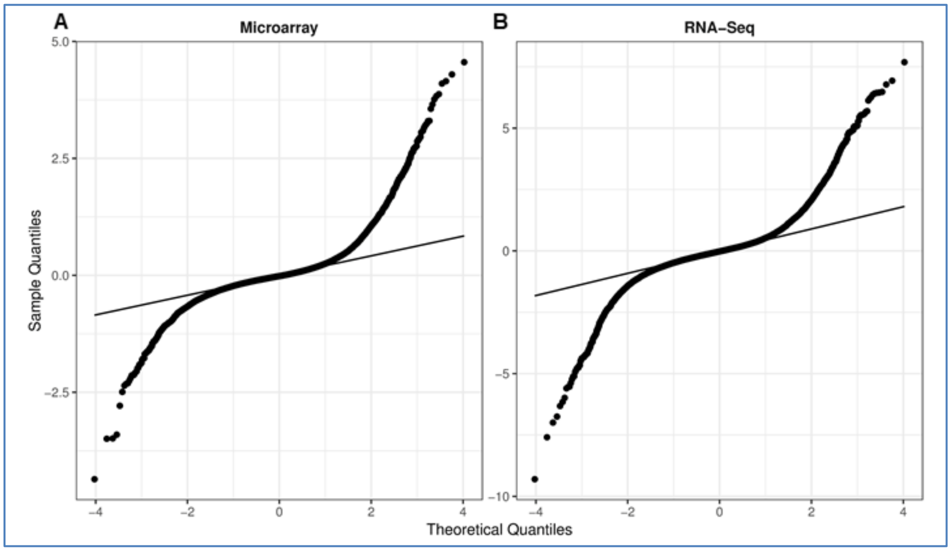

Figure S3. Normal Q-Q plots of the difference in region-specific gene effects using (A) microarrays and (B) RNA-Seq xpression data. There is evidence of extreme effects on both ends of all plots indicating the presence of differentially expressed genes.

Table S2. Parameter estimates of the two-component mixture model fitted to the difference (PUTM – SNIG) of region-specific gene effects. The probability that a gene is differentially expressed is comparable between microarrays and RNA-Seq models. The higher standard deviations from the RNA-Seq model are likely due to the higher REML-estimated variances.

Parameter	Microarray	RNA-Sequencing
$\hat{\pi}_1$	0.187	0.199
$\hat{\sigma}_0$	0.192	0.401
$\hat{\sigma}_1$	0.852	1.694
$\hat{\pi}_1$=probability that a gene is DE; $\hat{\sigma}_0$=SD of non-DE genes; $\hat{\sigma}_1$=SD of DE genes.		

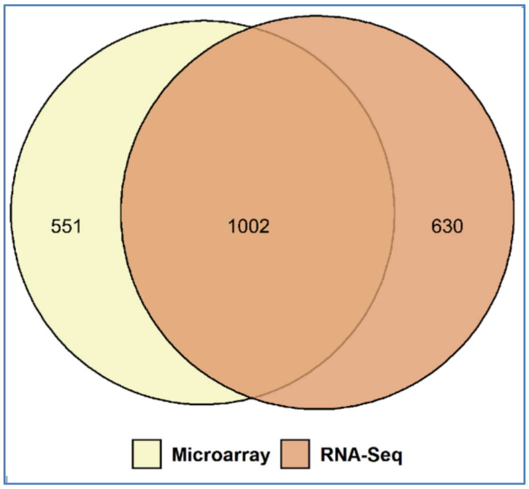

Figure S4. Significant DEGs identified by microarray expression data against RNA-Seq expression data. The total number of DEGs found are comparable between the technologies as seen by the similar areas of the Venn diagram, with 1002 DEGs in common.

Based on the distributions of (posterior) probabilities of genes being DE in both technologies, a threshold of 0.8 was used to declare DE genes ($\tau \geq 0.8$). This resulted in 1553 DEGs (8.7% of total genes) using microarray data, while using RNA-Seq data, 79 more DEGs were detected with a total of 1632 DEGs (9.3% of total genes) (Figure S4).

Expression QTL

As referred to in the main paper, there were 231 eQTLs identified by both platforms for PUTM and 27 for SNIG. The small number of eQTLs in common detected in SNIG may be a side effect of the smaller number of eQTLs found by using microarray data from SNIG. These proportions were similar even when mapping to haplotypes instead of individual SNPs and when using a less stringer FDR threshold of 0.05 (Figure S5 and S6).

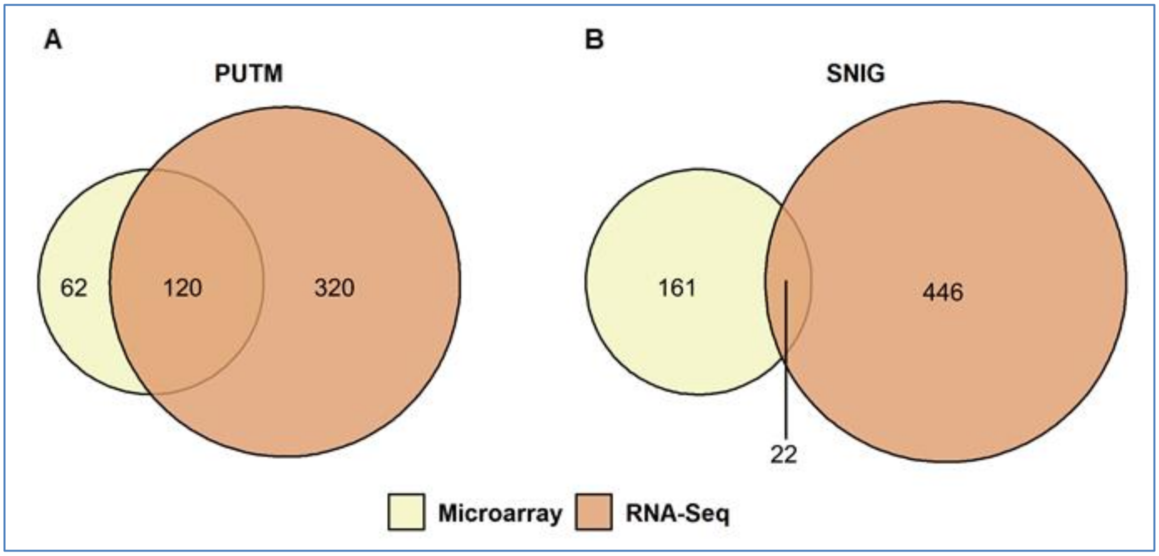

Figure S5. Significant eQTLs (FDR ≤ 0.01) mapped as haplotype to gene pairs (instead of SNP to gene) identified by microarrays against RNA-Seq in (A) PUTM and (B) SNIG. There is still a small overlap between technologies and the proportion of overlapped eQTLs remained as when mapped with SNPs (i.e. Figure 5).

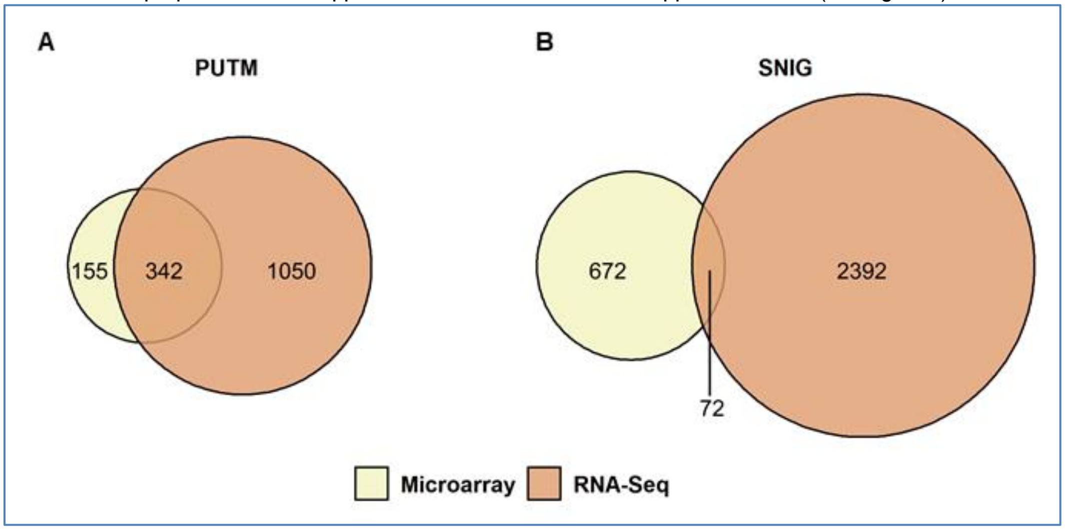

Figure S6. Significant eQTLs (FDR ≤ 0.05) identified by microarrays against RNA-Seq in (A) PUTM and (B) SNIG. There is still a small overlap between technologies and the proportion of overlapped eQTLs remain similar to using a more stringent FDR threshold (i.e. Figure S5).

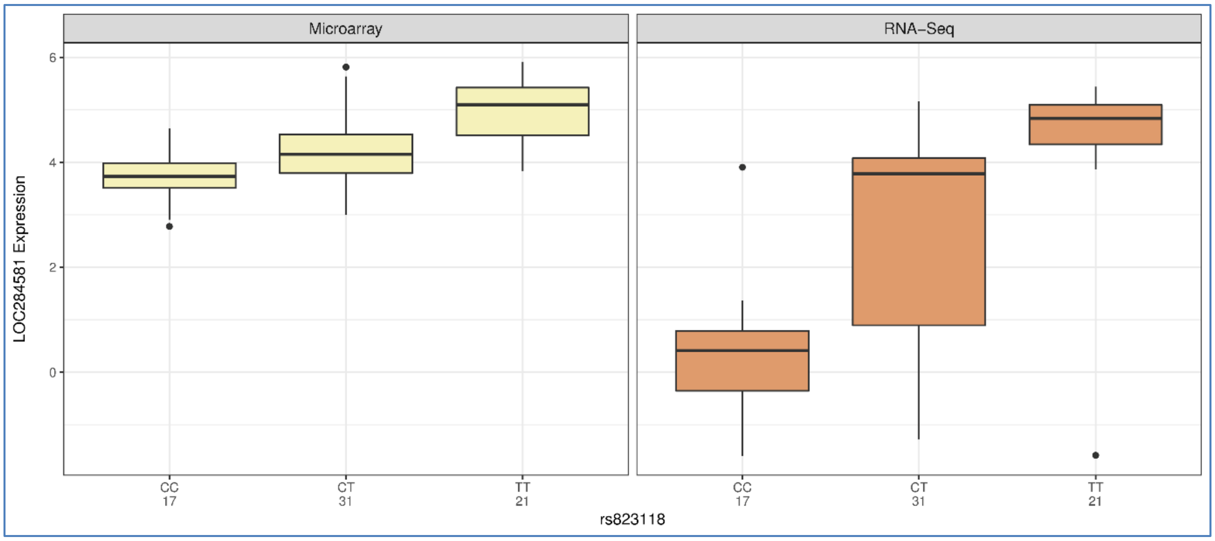

Figure S7. Boxplots of the effect of SNP rs823118 on LOC284581 expression levels in PUTM. The TT homozygous genotype is associated with an increase in LOC2854581 expression for both microarray and RNA-Seq data. Note that this eQTL was significant in PUTM only.

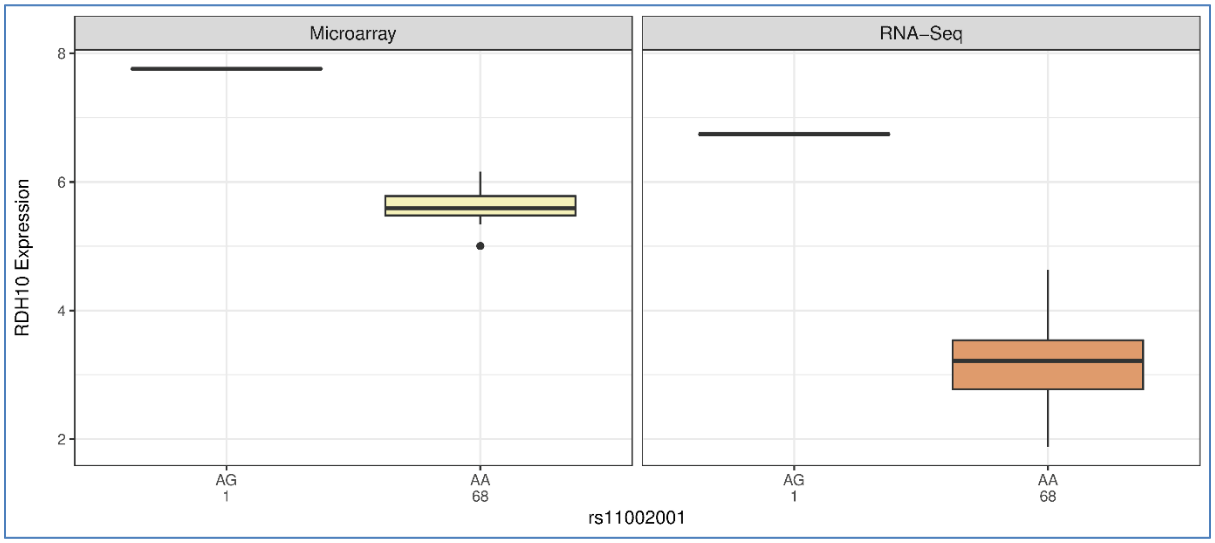

Figure S7. Boxplots of the effect of SNP rs11002001 on RDH10 expression levels in SNIG. The AA homozygous genotype is associated with a decrease in RDH10 expression for both microarray (P=1.28 × 10^{-14}) and RNA-Seq (P=3.21 × 10^{-10}) data. Note that this eQTL was significant in SNIG only and because of sample subsetting for platform comparison, there were no samples with the GG genotype. It is worth mentioning that the GTEx dataset had only one sample with the GG genotype out of 483 samples and in the 1000 Genome dataset, the G allele has a frequency of 0.0136).
